# A Leaderless Two-Peptide Bacteriocin, Enterocin DD14, Is Involved in Its Own Self-Immunity: Evidence and Insights

**DOI:** 10.3389/fbioe.2020.00644

**Published:** 2020-06-26

**Authors:** Rabia Ladjouzi, Anca Lucau-Danila, Abdellah Benachour, Djamel Drider

**Affiliations:** ^1^UMR Transfrontalière BioEcoAgro N° 1158, Univ. Lille, INRAE, Univ. Liège, UPJV, YNCREA, Univ. Artois, Univ. Littoral Côte d'Opale, ICV – Institut Charles Viollette, Lille, France; ^2^UR Risques Microbiens, Normandie Univ, UNICAEN, U2RM, Caen, France

**Keywords:** leaderless peptides, two-peptide enterocin DD14, bacterial self-immunity, regulation, antimicrobial peptide, ABC transporter

## Abstract

Enterocin DD14 (EntDD14) is a two-peptide leaderless bacteriocin produced by *Enterococcus faecalis* 14, a strain previously isolated from meconium. EntDD14 has a strong antibacterial activity against Gram-positive bacteria. Leaderless bacteriocins, unlike bacteriocins with leader peptides, are immediately active after their translation, and a producing strain has then to develop specific mechanisms to protect both intra and extracellular compartments. The *in silico* analysis of *Ent. faecalis* 14 genome allowed to locate downstream of structural *ddAB* genes, 8 other adjacent genes, designed *ddCDEFGHIJ*, which collectively may form three operons. To gain insights on immunity mechanisms of *Ent. faecalis* 14, mutant strains knocked out in *ddAB* genes encoding bacteriocin precursor peptides (Δ*bac*) and/or ABC transporter (Δ*ddI*) of EntDD14 were constructed and characterized. Importantly, Δ*bac* mutant strains, from which structural *ddAB* genes were deleted, resulted unable to produce EntDD14 and sensitive to exogenous EntDD14 showing their involvement in the *Ent. faecalis* 14 immunity system. Moreover, the sensitivity of Δ*bac* mutants appeared not to be associated with the down-regulation of *ddCDEFGHIJ* gene expression since they were similarly expressed in both Δ*bac* and wild-type strains during the log phase while they were found significantly down-regulated in the Δ*bac* mutant strain after 24 h of growth. Data gathered from this study suggest also the implication of the ABC transporter (*ddHIJ*) in the active export of EntDD14 but ruled-out its involvement in the primary self-immunity system. Interestingly, non-bacteriocin producing *Ent. faecalis* JH2-2 cells transformed with *ddAB*, or *ddAB* plus genes encoding the ABC transporter (*ddAB-HIJ*) did not produce EntDD14 and remained sensitive to its action. Of note, trans-complementation of the Δ*bac* mutant strain with these constructions allowed to recover the WT phenotype. To the best of our knowledge, this is the first study delineating the role of the intracellular two-peptide leaderless bacteriocins in their self-immunity.

## Introduction

Enterococci are members of the lactic acid bacteria (LAB) group that are known to produce potent bacteriocins, named enterocins. Bacteriocins are ribosomally synthesized, antimicrobial peptides active against a group of closely related species (Klaenhammer, 1993; Nes et al., 1996). Many studies have reported the promising potential of bacteriocins for use in in different areas like food industry (Perez et al., 2014; Juturu and Wu, 2018), and both clinical and veterinary applications (Papo and Shai, 2005; Diez-Gonzalez, 2007; van Heel et al., 2011; Hu et al., 2018). According to their physicochemical properties, bacteriocins can cause perturbations of cell membranes (pore formation, followed by efflux of metabolites and ions), depolarization of membranes, inhibition of cell wall synthesis or septum formation, disruption of DNA replication and transcription, or interference at the ribosomal level preventing protein synthesis (Cotter et al., 2013; Verma et al., 2014; Johnson et al., 2018).

Different classifications of bacteriocins have been proposed. Cotter et al. (2013) subdivided bacteriocins into two main classes. Class I contains bacteriocins that undergo significant post-translational modifications and class II contains unmodified peptides but that can undergo only slight modification such as the formation of disulphide bridges or circularization. Recently, Alvarez-Sieiro et al. proposed a classification constituted of three classes, with emphasis on modified bacteriocins (Alvarez-Sieiro et al., 2016). Class I contains small post-translationally modified peptides, designed RiPPs (ribosomally synthesized, post-translationally modified peptides), and are <10 kDa; class II contains unmodified bacteriocins smaller than 10 KDa; and class III contains unmodified bacteriocins larger than 10 KDa and endowed with bacteriolytic or non-lytic mechanisms.

Class II bacteriocins of the lactic acid bacteria can be divided into four groups; Class IIa pediocin-like bacteriocins are peptides that containing a YGNGV motif; Class IIb contain two-peptide bacteriocins whose antimicrobial activity depends on the complementarity of the two peptide partners; Class IIc are circular bacteriocins; and class IId groups unmodified linear peptides, non-pediocin-like, single-peptide bacteriocins that do not fit into the three other groups (Cotter et al., 2013). This latter group is relatively diverse and includes a leaderless bacteriocin subgroup whose members are different from other bacteriocins. While most bacteriocins are synthesized with an N-terminal leader peptide, leaderless bacteriocins do not involve any N-terminal leader sequence for their export outside of the cell (Cotter et al., 2013).

Many leaderless bacteriocins with different characteristics have been recently described in the literature, overall constituting a large group. Leaderless bacteriocins can be composed of from one to four peptides. The leaderless single peptide bacteriocins include enterocin Q, aureocin A53, BHT-B, LsbB, lacticin Q, lacticin Z, weisselicin Y, weissellicin M, enterocin EJ97, enterocin K1, epidermicin NI01, lactolisterin BU, for review see Perez et al. (2018). The leaderless two-peptide bacteriocins group enterocin L50 (A, B), enterocin MR10 (A, B), enterocin DD14 (A, B), enterocin Ent7 (A, B) and enterocin A5-11 (A, B) (Cintas et al., 1998; Batdorj et al., 2006; Martín-Platero et al., 2006; Liu et al., 2011; Caly et al., 2017). The leaderless multi-peptide bacteriocins include aureocin A70 (A, B, C, D) (Netz et al., 2001) and garvicin KS (A, B, C), cereucin X (A, B, C), cereucin V (A, B, C), and cereucin H (A, B, C, D) (Ovchinnikov et al., 2016).

Enterocin DD14 is a two-peptide leaderless bacteriocin produced by *Ent. faecalis* 14 isolated from meconium at the Roubaix Hospital, in the north of France (Al Atya et al., 2015). The antagonistic activity of EntDD14 was reported against a panel of Gram-positive bacteria such as Staphylococcus aureus, Listeria monocytogenes, *Ent. faecalis*, Bacillus subtilis and *Clostridium perfringens*.

The scale-up production of bacteriocins requires an understanding of their genetic regulation and expression. Thus, it would be pertinent to identify the mechanisms involved in the secretion of EntDD14, a bacteriocin which lacks a transport signal domain. It has been reported that bacteriocin production is generally associated with the expression of specific immunity proteins, allowing protection of the bacteriocinogenic strain from the toxicity of its own bacteriocin (Bierbaum and Sahl, 2009; Nissen-Meyer et al., 2009). The leaderless bacteriocins are active immediately after their translation process (Perez et al., 2018). Their intracellular toxicity has been reported for lacticin Q (Iwatani et al., 2012), but many questions related to this topic remain to be solved. The self-immunity system would have to protect the bacteriocinogenic strain from both intra- and extracellular bacteriocins. To date there are little data in the literature reporting biosynthetic pathways of leaderless bacteriocins. Genes implicated in the secretion and immunity of aureocin A70 (Netz et al., 2001; Coelho et al., 2014), aureocin A53 (Nascimento et al., 2012) and lacticins Q and Z (Iwatani et al., 2012, 2013) have been experimentally confirmed. Interestingly, the protein LmrB, an ABC-type multi-drug transporter, has been reported to play a role in secretion and immunity of the leaderless bacteriocin, LsbB, and that of a non-leaderless bacteriocin, LsbA (Gajic et al., 2003). Here, the two-peptide leaderless bacteriocin EntDD14 has been investigated at the molecular level to decipher and better understand its role in the self-immunity system.

## Materials and Methods

### Bacterial Strains, Plasmids, and Growth Conditions

Bacterial strains, plasmids and oligonucleotides used in this study are listed in Tables 1, 2. Cultures of *Ent. faecalis, Listeria innocua* and *C. perfringens* strains were performed in M17 medium supplemented with 0.5% (w/v) of glucose (GM17) or in brain heart infusion (BHI) at 37°C under semi-aerobic or anaerobic conditions. Where necessary, media were supplemented with erythromycin (Em) (150 μg/mL) or chloramphenicol (Cm) (15 μg/mL). Growth of the cultures was followed by measuring the optical density at a wavelength of 600 nm (OD_600_) with a spectrophotometer (Aquoalabo, France) and by determining cfu counts on agar plates. *Escherichia coli* strains were cultivated at 37°C in Luria-Bertani (LB) broth with vigorous shaking (160 rpm) or on LB agar medium. Where appropriate, ampicillin (100 μg/mL), erythromycin (150 μg/mL) or chloramphenicol (10 μg/mL) was added to the medium. Bacterial stocks were stored at −80°C in GM17, BHI or LB broth supplemented with 50% (v/v) glycerol.

**Table 1:** Bacterial strains and plasmids used in this study.

**Designation**	**Relevant characteristics**	**Reference**
**Strain**		
***Enterococcus faecalis***		
14	Wild-type strain isolated from meconium	Al Atya et al., 2015
14 *Δbac*	*Ent. faecalis* 14 *ddA* and *ddB* deletion mutant	This study
14 *Δbac-Comp*	*Ent. faecalis* 14*ΔddA/ddB* harboring pAT18:*ddA/ddB*	This study
14 Δ*ddI*	*Ent. faecalis* 14 *ΔddI gene* deletion mutant	This study
14 Δ*ddI-Comp*	*Ent. faecalis* 14 *ΔddI* harboring pAT18:*ddI*	This study
14 *ΔddIΔbac*	*Ent. faecalis* 14 *ΔddI, ΔddA* and *ΔddB* deletion mutant	This study
14 *ΔddIΔbac-Comp*	*Ent. faecalis* 14*ΔddI ΔddA/ddB* harboring pAT18:*ddAB-HIJ*	This study
JH2-2	Fus^R^ Rif^R^ plasmid-free wild-type strain	Yagi and Clewell, 1980
JH2-2-*ddAB*	*Ent. faecalis* JH2-2 harboring pAT18:*ddA/ddB* of *Ent. faecalis* 14	This study
JH2-2-*ddAB-HIJ*	*Ent. faecalis* JH2-2 harboring pAT18:*ddAB-HIJ* of *Ent. faecalis* 14	This study
V583	Clinical isolate from blood. Van^R^, Em^R^	Sahm et al., 1989
***Listeria innocua***	*Listeria innocua* ATCC 33090	Bougherra et al., 2017
***Clostridium perfringens***	DSM756 corresponds to the type strainATCC®13124^TM^ (type A, α+)	DSM
***E. coli***		
XL-1 Blue	*recA1 endA1 gyrA96 thi-1 hsdR17 supE44 relA1 Lac* [F' *proAB lacI* ^q^ZΔM15Tn*10* (Tet^R^)]	Stratagene
JM109	*recA1. endAl, gyrA96, thi. hsdR17, supE44, relA1, I-, A(iac-proAB), [F', traD36, proAB, iacI^*q*^ZAM15]*	Yanisch-Perron et al., 1985
**Plasmids**		
pLT06	*lacZ*, P-*pheS* from pCJK47, *Cm* from pGB354, *orfB, orfC, repA* (*Ts*), *orfD* from pCASPER	Thurlow et al., 2009
pLT06*ΔddAB*	pLT06 derivative carrying a 2.7 kb DNA fragment from *Ent. faecalis 14* harboring mutated *ddA* and *ddB* gene	This study
pLT06*ΔddI*	pLT06 derivative carrying a 2.139 kb DNA fragment from *Ent. faecalis 14* harboring mutated *ddI* gene	This study
pAT18	Em^R^, Tra ^−^, Mob ^+^, *Iac*Za^+^, *ori*R pUC, *ori*R pAMβI, MCS pUCl8	Trieu-Cuot et al., 1991
pAT18:*ddAB*	pAT18 derivative carrying a 0.64 kb DNA fragment from *Ent. faecalis* 14 with *ddA and ddB genes*	This study
pAT18:*ddI*	pAT18 derivative carrying a 0.765 kb DNA fragment from *Ent. faecalis* 14 with *ddI genes*	This study
pAT18:*ddAB-HIJ*	pAT18 derivative carrying a 1.43 kb DNA fragment from *Ent. faecalis* 14 with *ddAB* and *ddHIJ genes*	This study

*Amp, ampicillin; Em, erythromycin; Fus, fusidic acid; Rif, rifampin; Str, streptomycin; Kan, kanamycin; Tet, tetracycline; Cm, chloramphenicol; bgaB, Beta-galactosidase; R, resistant; S, sensitive; Ts, thermosensitive*.

**Table 2:** Oligonucleotides used in this study.

**Name**	**Sequence 5^**′**^ → 3^**′**^**	**Use**
**Construction of** ***Ent. faecalis*** **14** ***Δbac mutant*** **and complementation**		
Bac1F -PstI	AAAAAACTGCAGGAGGAACCCTTATTTTAAAGGATTC	Amplification of *ddA/ddB* up-stream fragment
Bac2R-Stop	CATTCACTAGGATCCTTAGACTTACTCCCATAATATATCTCCTCC	
Bac3F-Stop	TAAGTCTAAGGATCCTAGTGAATGGGTTAAAAAGACATTGATTTT	Amplification of *ddA/ddB* down-stream fragment
Bac4R -NcoI	AAAAAACCATGGCCGTGTATACTTTAGCCTTAGG	
Bac5F	CGGTGGATTATGAGACTGGAAC	Outer primer; verification of the plasmid integration
Bac6R	GCTCGATTTTTTTCCAATATTT	
BacF-screen	GTCTAAGGATCCTAGTGAATG	Screening of the mutants
Bac-compF-KpnI	AAAAGGTACCATCATGTTGATGACTAGAATCC	Complementation of *Δbac* mutant
Bac-compR-BamHI	AAAAGGATCCAAGGATATACTTATTATTTCACAG	
ORIf	CAATAATCGCATCCGATTGC	Cloning verification in pLT06 plasmid
KS05seqR	CCTATTATACCATATTTTGGAC	
**Construction of** ***Ent. faecalis*** **14** ***ΔddI*** **mutant and complementation**		
ddI 1F-PstI	ATTAAACTGCAGCGCCACTGAGCCAAAAGAAG	Amplification of *ddI* up-stream fragment
ddI 2R-Stop	CATTCACTAGGATCCTTAGACTTAGAGTTTAATCATTGTTTCACCG	
ddI 3F-Stop	TAAGTCTAAGGATCCTAGTGAATGGAATCTTTATGTAAACATGCG	Amplification of *ddI* down-stream fragment
ddI 4R-NcoI	ATTAAACCATGGCCTTCTAAAAGGAATTGTAAC	
ddI 5F	GGCGCGACCCTTATTTATAAG	Outer primer; verification of the plasmid integration
ddI 6R	GTGAACCTTTATCAAGGAGCC	
ddI -compF-BamHI	ATTAAAGGATCCGACTGGACTTAAAGAAAATGATTC	Complementation of *ΔddI* mutant
ddI -compR-PstI	ATTAAACTGCAGCCTATCATGGTTAATACACTACG	
**Construction of pAT18:*****ddAB-HIJ***		
Op AF-KpnI	AAAAGGTACCATCATGTTGATGACTAGAATCC	Amplification of *ddA* and *ddB*
Op BR	A C C G A C C T G C A G T A A A C C G A C A A C AAGGATATACTTATTATTTCACAG	
Op HF	G T T G T C G G T T T A C T G C A G G T C G G TGATATAGGAGAAGATAATGAGTAA	Amplification of ABC transporter *ddHIJ*
Op JR-BamHI	AAAAGGATCCATGTGACAGCCTGTCTAATTC	
PU	GTAAAACGACGGCCAGT	Cloning verification in pAT18 plasmid
PR	CAGGAAACAGCTATGAC	

*Underlined sequences correspond to the recognition sites of the restriction endonucleases mentioned in the primer name. The sequences underlined by a discontinuous line represent complementary sequences*.

### General Molecular Methods

Molecular cloning and other standard techniques were performed essentially as previously described (Sambrook and Russell, 2001). Antibiotics, chemicals, and enzymes were of reagent grade. Restriction endonucleases and T4 ligase were obtained from ThermoFicher Scientific and used in accordance with the manufacturer's instructions. Plasmids and PCR products were purified using NucleoSpin kits (Macherey-Nagel, Dünen, Germany) and the final plasmid constructions and the resulting deletion mutants were verified by PCR and sequencing (Eurofins, Ebersberg, Germany). The resulting sequences were analyzed using the SnapGenes tool (GSL Biotech LLC, CA). The strains were transformed by heat shock for *E. coli* and by electroporation for *Ent. faecalis* using Gene Pulser Apparatus (Bio-Rad Laboratories).

### Antibacterial Activity Assays

The screening of antimicrobial activity of WT *Ent. faecalis* 14 and its isogenic mutant strains against the target bacteria was performed using well-diffusion and the spot-on-lawn methods (Caly et al., 2017). Briefly, BHI plates (1% agar) were inoculated with *L. innocua, C. perfringens, Ent. faecalis* JH2-2 and *Ent. faecalis* V583 strains and were allowed to dry. Then, 4 μL of a bacterial culture or 50 μL of culture supernatant/pure EntDD14 of the tested strain was spotted on the plate and incubated overnight at 37°C in appropriate conditions. The radius of the inhibition zone was measured from the edge of the well/spot to the edge of the inhibition halo. Antibacterial activity was quantified using the Arbitrary Units (AU) method (Cintas et al., 2000).

### EntDD14 Purification and Quantification

A volume of 200 mL culture of *Ent. faecalis* 14 was grown for 24 h in GM17 medium at 37°C without shaking. The culture supernatant was harvested by centrifugation (8,000 rpm) and was incubated for 24 h at 4°C with CM Sephadex® C-25 (GE Healthcare Life Sciences, Milwaukee, WI). The resin was then washed with 5 bed volumes (BV) of 50 mM sodium phosphate (pH 6.3) and 5 BV of 0.5 M NaCl. The resin-bound bacteriocin was then eluted with 2 BV of 1.5 M NaCl. The EntDD14 was then further purified by gel filtration using PD MidiTrap G-10 columns (GE Healthcare Life Sciences) in Milli-Q water. The eluted solution was dried by miVac Sample Concentrators (SP Scientific NY-USA) and the dried samples were then resuspended in appropriate volumes of Milli-Q water to give the desired concentration. After each purification step, protein concentration was measured using a bicinchoninic acid assay (Sigma-Aldrich). The purity of the EntDD14 was verified by MALDI-TOF/MS analyses (Bruker Daltonics, Bremen, Germany) according to the recommendations of the manufacturer.

### Sensitivity Assays to Pure EntDD14

To analyze the sensitivity of the *Ent. faecalis* 14 mutant strains, and other targets to the purified EntDD14, kinetics at 37°C in GM17 medium, supplemented with purified EntDD14 at a final concentration of 10 μg/mL, were performed in a 96-well microplate using a SpectraMax i3 spectrophotometer (Molecular Devices, CA, USA). The cultures were inoculated with the same bacterial charge thus giving the same initial OD_600_ of 0.2. Measurements were taken every 30 min at OD_600_ for 12 H. In parallel, the activity of EntDD14 (60 μg/mL) or serial dilutions of culture supernatant was also tested using the well-diffusion assay and the bacteriocin activity was expressed in AU/mL, which corresponds to the inverse of the last active dilution multiplied by 100 (Batdorj et al., 2006).

### Construction of the Double Mutant Strain *Δbac*

The double mutant strain Δ*bac* was constructed by allelic exchange using a method based on the conditional replication of the pLT06 vector (Thurlow et al., 2009). For this purpose, we used the *Ent. faecalis* 14 strain (Al Atya et al., 2015) to prepare the DNA template for PCR amplification using the Phusion™ High-Fidelity DNA Polymerase Mix (Thermo Fisher Scientific, Strasbourg, France). First, we amplified separately the upstream (900 bp) and downstream (1,057 bp) regions of the *ddA/ddB* genes using the primer pairs Bac1F-PstI/Bac2R-Stop and Bac3F-Stop/ Bac4R-NcoI, respectively (Table 2). The Bac3F-Stop and Bac2R-Stop primers share a complementary sequence of 24 bp, where four stop codons and the BamHI restriction site were inserted (Figure S1). To generate the entire DNA fragment harboring the mutated *ddA* and *ddB* genes, a second PCR was performed using the primer pair Bac1F-PstI/Bac4R-NcoI and the mix of the two previous amplified fragments as template DNA. The resulting DNA fragment of 1,981 bp was excised from the electrophoresis agarose gel and purified. After digestion with appropriate restriction enzymes (*Pst*I and *Nco*I), this fragment was cloned into the pLT06 plasmid to generate pLT06Δ*ddAB* using the *E. coli* JM109 strain. This recombinant plasmid was used to transform *Ent. faecalis* 14 and clones were obtained on GM17 with 15 μg/mL of Cm at 30°C (the permissive temperature of the plasmid). To trigger the first cross-over (CO), cultures were grown for 2½ h at 30°C then shifted to 42°C for an additional 4 h in the presence of Cm. After serial dilutions, agar plates supplemented with 15 μg/mL Cm and X-Gal 80 μg/ml were inoculated. The larger blue colonies were verified by PCR using the outer primers Bac5F with one of the primers located on the plasmid (ORIf or KS05seqR). A colony from the 1st CO was cultured in the absence of selection pressure until the culture had reached stationary phase (~2 × 10^9^ cfu/mL). Serial dilutions were prepared and a new culture of ~100 cfu/mL was inoculated and subsequently grown to stationary phase overnight. At this step, the chloramphenicol-susceptible clones underwent the 2nd CO and were screened for the deletion of the *ddA* and *ddB* genes. The BacFscreen primer (Table 2) was designed on the basis of the introduced modified sequence in place of the *ddA* and *ddB* sequence (Figure S1) thereby allowing the screening by PCR of the chloramphenicol-susceptible clones obtained after the second crossing-over event. When BacFscreen primer was used in combination with the Bac6R primer (located outside of the construct; Figure S1), only the double mutant strain Δ*bac* clones were able to produce a 1,132 pb DNA product. Moreover, PCR with Bac5F/Bac6R amplified a shorter fragment than that amplified in the wild-type, confirming that the deletion occurred in the structure of the *ddA* and *ddB* genes. Finally, the EntDD14 genetic environment of the obtained mutant clones was also verified by sequencing.

### Construction of the *ΔddI* Mutant Strain

To delete the gene *ddI* of *Ent. faecalis*14 corresponding to an ATP-binding protein of the ABC transporter, a strategy identical to that used to generate the Δ*bac* mutant strain was followed. Briefly, the upstream (1,128 bp) and downstream (987 bp) regions of the *ddI* gene were amplified using the primer pairs ddI 1F-PstI/ddI 2R-Stop and ddI 3F-Stop/ddI 4R-NcoI, respectively (Table 2). A second PCR using the primer pair *ddI* 1F-PstI/*ddI* 4R-NcoI was performed using the mix of the two previous amplified fragments as DNA template in order to obtain the entire DNA fragment harboring the mutated *ddI* gene. After that, the resulting DNA fragment was cloned into the pLT06 vector and the resulting plasmid pLT06Δ*ddI* was transformed into *E. coli* JM109 then into *Ent. faecalis* 14. After the 1st and 2nd CO events, the chloramphenicol-susceptible clones were screened for the deletion of *ddI* gene by PCR using BacFscreen/ddI 6R and ddI 5F/ddI 6R primer pairs. Finally, the genetic environment of the resulting mutant strains was verified by sequencing.

### Construction of the Triple Mutant Strain *ΔI*Δ*bac*

The Δ*ddI* mutant strain was used to generate the *Ent. faecalis* 14 Δ*I*Δ*bac* triple mutant strain by using a double homologous recombination as described above. Competent *Ent. faecalis* 14 Δ*ddI* cells were transformed by the recombinant pLT06Δ*ddAB* plasmid and the same method was then used to obtain the triple mutant strain Δ*I*Δ*bac*. All these mutant strains and their required genetic correctness were checked by PCR and sequencing.

### Complementation of *Ent. Faecalis* 14 Mutant Strains and Construction of *Ent. Faecalis* JH2-2 Harboring Structural and ABC Transporter Genes

For the complementation assays, a DNA fragment containing the entire *ddA, ddB* and the promoter region was obtained by PCR using Bac-compF-KpnI and Bac-compR-BamHI primers and cloned into pAT18 (Table 1). The recombinant plasmid pAT18:*ddAB* was used to transform competent cells of the Ent. faecalis 14 Δ*bac* mutant and Ent. faecalis JH2-2 after passing through *E. coli* XL-1 blue selected on LB, Em (150 μg/mL), X-gal (40 μg/mL) and IPTG (40 μg/mL). The colonies obtained on GM17 agar Em (150 μg/mL) after 48 h at 37°C, (Ent. faecalis 14Δ*bac comp* and JH2-2 pAT18:*ddAB*) were analyzed by PCR and sequencing for the presence of the recombinant plasmid. In the same way we complemented the Δ*ddI* mutant strain using the primers *ddI*-compF-BamHI and *ddI*-compR-PstI. To complement the Δ*I*Δ*bac* triple mutant strain, we constructed a pAT18:*ddAB-HIJ* containing the structural genes *ddA* and *ddB* and the *ABC* transporter module *ddH, ddI*, and *ddJ* intact genes. For this, a first PCR with the OpAF-KpnI/OpBR primer pair was used to amplify the *ddAB* genes including the promoter region. A second PCR with the Op HF/Op JR-BamHI primers amplified the *ddHIJ* genes of the ABC transporter. As the Op BR and Op HF primers share a complemented sequence of 24 bp (see Table 2), a PCR using the AF-*Kpn*I/Op JR-*Bam*HI primers was able to amplify the *ddAB-HIJ* genes. After digestion by *Bam*HI/*Kpn*I, the obtained fragment was cloned into the pAT18 vector to obtain pAT18:*ddAB-HIJ* (3,7 kb). This construction was used to transform the Δ*I*Δ*bac* mutant and *Ent. faecalis* JH2-2 strains leading to Δ*I*Δ*bac*-*Comp* and *Ent. faecalis* JH2-2-*ddAB-HIJ* strains, respectively.

### RNA Isolation and Microarrays Analysis

For microarray analysis, three distinct cultures of *Ent. faecalis* 14Δ*bac* were used (Δ*bac1*, Δ*bac2* and Δ*bac3*) in order to compare with the *Ent. faecalis* 14 wild-type strain (WT1, WT2 and WT3), after 6 and 24 h of growth in GM17 medium. Analyses were performed with total RNA isolated using NucleoSpin^TM^ RNA Plus columns (Macherey-Nagel, Hoerdt, France). RNA quality was verified with Nanodrop and absorbance ratios A260/280 and A260/230 were between 2.0 and 2.2. RNA quality was also examined with Bioanalyzer 2100 (Agilent, Les Ulis, France) and a minimal RNA integrity number (RIN) of 0.8 was required for all samples.

Agilent G2509F *Ent. faecalis* custom oligo-based DNA microarray (8 × 15 K) containing spots of 60-mer oligonucleotide probes (in duplicate) were used to study the gene expression profile. RNA amplification, staining, hybridization and washing were conducted according to the manufacturer's instructions. Slides were scanned at 5 μm/pixel resolution using the GenePix 4000B scanner (Molecular Devices Corporation, Sunnyvale, CA, USA). Images were used for grid alignment and expression data digitization with GenePix Pro 6.0 software. Expression data were normalized by Quantile algorithm. To ascertain the quality of normalized data, filtering of data was mandatory for flagged signals. Expression data from the 3 wild-type samples were filtered for *p* < 0.05 and the average was calculated for each gene. Genes of interest were selected for this study (*ddABCDEFGHIJ*) and the log2 ratio from individual Δ*bac* samples and the mean of WT samples was calculated. The corresponding probes designed for these genes are shown in Table 3.

**Table 3:** The probes designed for microarray experiments.

**Target gene**	**Probe sequence**
*ddA*	GCAATTTATTGGAGAAGGATGGGCAATTAACAAAATTATTGATTGGATCAAAAAACATAT
*ddB*	ACAAACAAATTATGCAGTTTATCGGACAAGGATGGACAATAGATCAAATTGAAAAATGGT
*ddC*	AAAGCAAGGTATTCACAAGATTCTTTAAAAGAGTATTTAGATATTCTTGTTTCTGAGGAT
*ddD*	TGAGTTTGAATGTAATTGTGTTGTTAGCGCTATTAGCTGTGCAAACGTGGCTGATACCAA
*ddE*	AGGTTCAATTCTAGTTATAGGCATTGCAGGTTTGGTTCAAACCATTATTGATCAAAAATT
*ddF*	ACGTGGGGTTACTGAGACACAAGTATCAAAAGTTTTTAAGGAGTTAAATAATAAAGAGAA
*ddG*	ACCCGGATCGCTCAATCCAGATAAAACTTTTTATCAAAAACTTGGCAATGATGGAATGGA
*ddH*	AAAAGCAACAGACGGAATAATTACTGAAATTAACGCACTGCCTGAAGAGATGGCTGTCAG
*ddI*	TAACGCTTATTATGGTTACGCACAACCCTGAAGTCGTTCCTTATTGTCACCGGTTGATTA
*ddJ*	TTATTAGCAGTTGGCGTCTCTTCAGTTATTGGCCTAGTTTTCTCTGTAATGCCTGCATCA

### Bioinformatics Analysis

The *Ent. faecalis* 14 genome sequence was obtained from GenBank under accession number CP021161. Here, different databases and bioinformatics tools were used to analyze the DNA sequences and predict the EntDD14 operon organization, with location of putative promoters and terminators. This analysis included the Basic Local Alignment Search Tool of the National Center for Biotechnology Information (BLAST-NCBI: https://blast.ncbi.nlm.nih.gov/Blast.cgi), the SnapGene®4.3.7 tool, the Softberry software for analysis of bacterial genomes (http://www.softberry.com), the ARNold program (http://rssf.i2bc.paris-saclay.fr/toolbox/arnold/index.php#Results) and the RibEx program (Abreu-Goodger and Merino, 2005)

### Statistical Analysis

Differences between samples were calculated using the Student test. *P* ≤ 0.05 were considered to be significant.

## Results

### Genetic Environment of EntDD14 Encoding DNA

**Figure 1:**
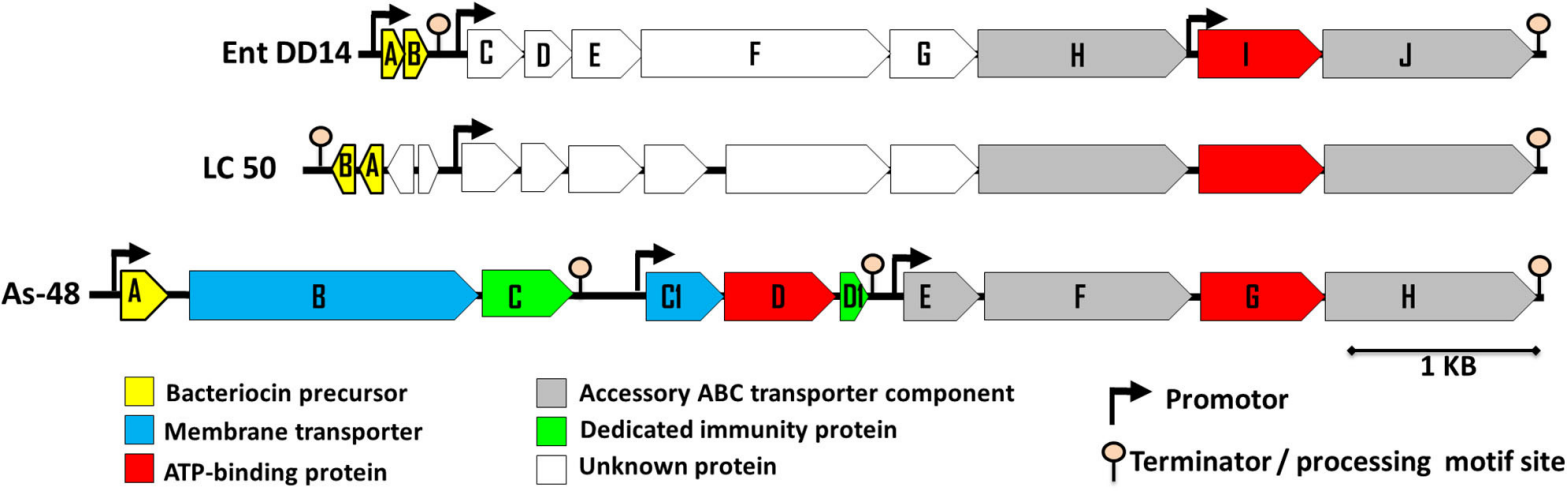
Genetic organization of *Ent. faecalis* 14 predicted ORFs. The genetic organization of *Ent. faecalis* 14 is compared to that of *Ent. faecalis* LC50 and to that of *Ent. faecalis* AS48 that are shown on the figure and produce enterocins L50AB and AS-48, respectively. The predicted promoters and ORFs were obtained using *Softberry* software.

**Figure 2:**
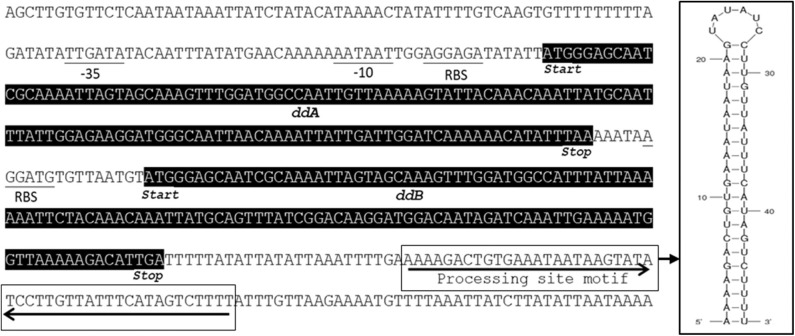
Genetic characterization of the *ddA* and *ddB* structural genes. The processing site motif has been detected by using both ARNold and RibEX programs.

**Figure 3:**
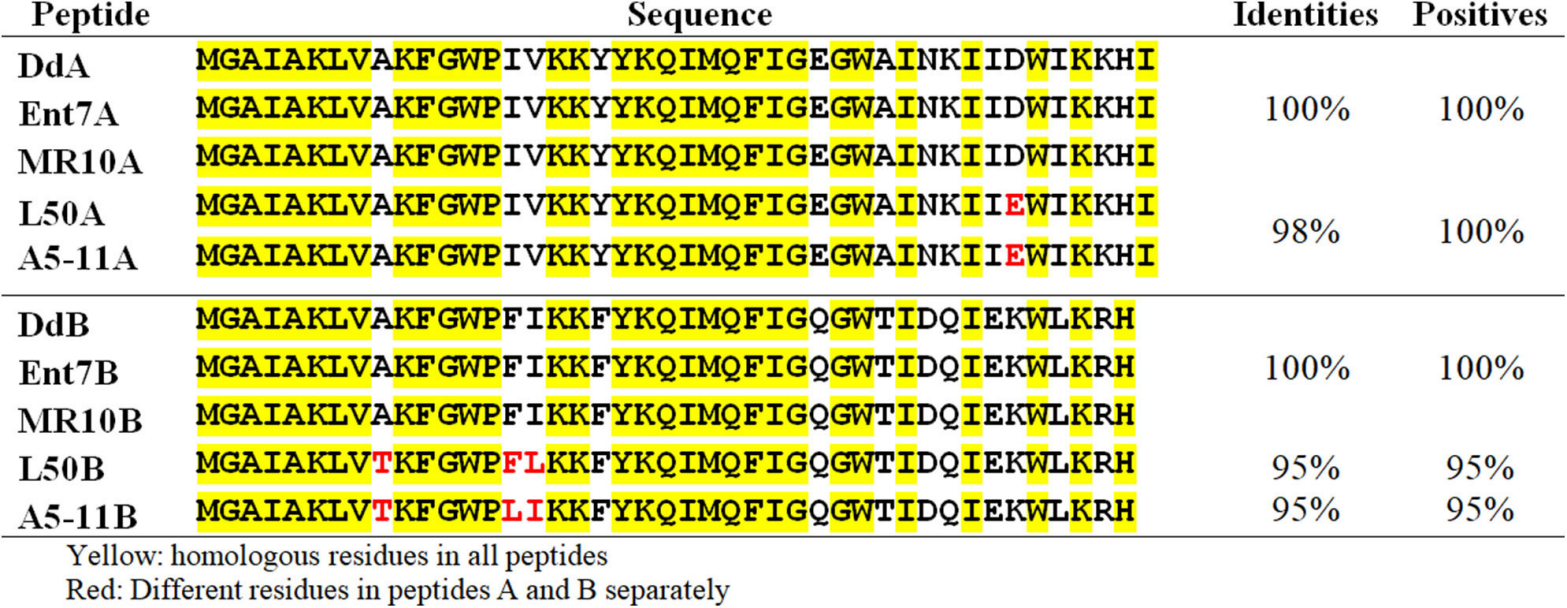
Alignment of the EntDD14 peptides with other leaderless two-peptide bacteriocins. The EntDD14 peptides (A and B) are aligned with the corresponding peptides of Ent 7, MR-10, L50, and A5-11 produced by *Ent. faecalis* 710C, *Ent. faecalis* MRR 10-3, *Ent. faecium* L50, and *Ent. Durans*, respectively.

Previous sequencing of the *Ent. faecalis* 14 genome showed that the EntDD14 encoding genes are chromosomal determinants (Belguesmia et al., 2017). *In silico* analysis of the genetic neighborhood of the EntDD14 structural genes revealed a cluster of 10 open reading frames (ORFs) *ddABCDEFGHIJ* (Figure 1). This cluster encodes two-peptide leaderless bacteriocins (DdA and DdB), five unknown proteins (DdC, D, E, F, and G) and a putative ABC transporter (DdH, I and J) (Table 4). The sequence alignment of this 6.5kb region with the BLAST-NCBI showed a high homology with the translated two-peptide leaderless bacteriocin regions of other known enterococci such as *Ent. faecalis* DBH18 (GenBank ID: EF502034.2) (Arbulu et al., 2016), *Ent. faecalis* KB1 (GenBank ID: CP022712.1), Ent. faecalis plasmid Efsorialis-p1 (GenBank ID: CP015884.1), *Ent. faecium* SRCM103341 plasmid (GenBank ID: CP035137.1), *Ent. faecium* strain FA3 plasmid (GenBank ID: CP042833.1), *Ent. faecium* strain 6T1a plasmid pEF1 (GenBank ID: DQ198088.1), *Ent. faecium* strain HB-1 plasmid (GenBank ID: CP040877.1), *Ent. faecium* strain SRCM103470 plasmid (GenBank ID: CP035221.1), *Ent. faecium* strain Gr17 plasmid pGR17 (GenBank ID: CP033377.1) and *Ent. faecium* strain 4928STDY7387800 (GenBank ID: LR607382.1). Noteworthy, the sequence analysis by the *Bacterial Operon and Gene Prediction* (http://www.softberry.com) predicted three operons. The first operon consisted of the *ddAB* genes, the second contained the *ddCDEFGH* genes and the third consisted of the two *ddIJ* genes (Figure 1). The structural genes *ddA* and *ddB* are expected to be co-transcribed as only one promoter has been detected upstream of the *ddA* gene and a clear processing site motif of 48 bp was detected between the *ddB* and *ddC* genes (Figure 2). The alignment of the DdA and DdB peptides with all other known two-peptide leaderless bacteriocins displayed a very high degree of identity. In this sense, EntDD14 was identical to MR-10 and Ent7 produced by *Ent. faecalis* MRR 10-3 (Martín-Platero et al., 2006) and *Ent. faecalis* 710C (Liu et al., 2011), respectively (Figure 3). Moreover, DdA and DdB were 98 and 95% identical to the corresponding peptide of L50 and A5-11 bacteriocins produced, respectively, by *Ent. faecium* L50 (Cintas et al., 2000) and *Ent. durans* (Batdorj et al., 2006; Belguesmia et al., 2013). Interestingly, all the bacteriocinogenic strains above-cited share at least a set of 3 genes, which are highly homologous to the second ABC transport system (As-48FGH) described for the cyclic bacteriocin AS-48 (Diaz et al., 2003) (Figure 1).

**Table 4:** Characteristics of the predicted ORF products of DD14 gene *locus*.

***ORF***	**Size (aa)**	**PI**	**MW**	**TM**	**Average of hydrophobicity**	**Characteristics of encoded protein**
*ORF A*	44	10	5176.35	0	0.202273	Peptide A of two-peptide leaderless bacteriocin
*ORF B*	43	10.22	5182.27	0	−0.109302	Peptide B of two-peptide leaderless bacteriocin
*ORF C*	89	8.57	10578.27	0	−0.555056	DUF2089 family protein. Soluble. Unknown function
*ORF D*	90	9.70	0702.05	2/3^*^	0.748889	Membrane protein. Unknown function
*ORF E*	135	9.54	15998.46	2	0.548148	Membrane protein YdbT. contains bPH2 domain
*ORF F*	458	9.43	54950.64	7/6^*^	0.281441	Membrane protein. PH domain-containing protein
*ORF G*	163	8.95	18588.34	4	0.668712	Membrane protein. Unknown function
*ORF H*	406	5.88	45035.10	1	−0.547044	Periplasmic component of efflux system
*ORF I*	227	6.10	25716.70	0	−0.192952	ABC transporter ATP-binding protein. ATPase
*ORF J*	399	9.15	44043.23	4	0.044361	ABC transporter. FtsX-like permease family protein

* *Result obtained using TMHMM program (http://www.cbs.dtu.dk/services/TMHMM/)*.

### Antimicrobial Activity of *Ent. Faecalis* 14 Is Solely Due to EntDD14

**Figure 4:**
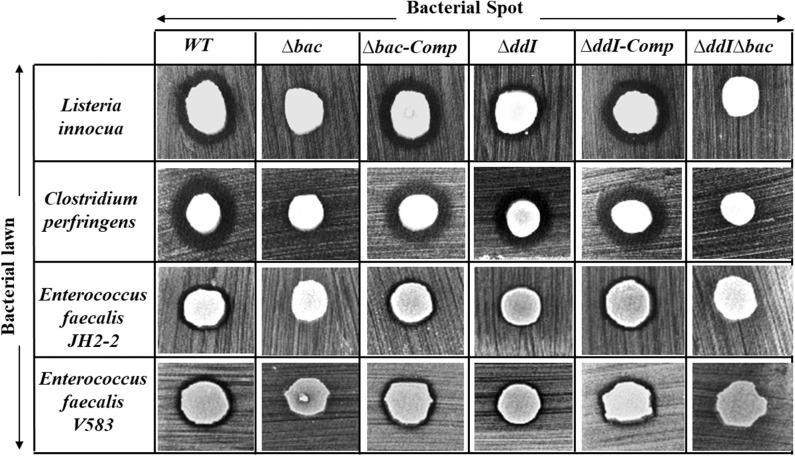
Antimicrobial activity of *Ent. faecalis* 14 wild-type, its null and complemented mutant strains against *Listeria innocua, Clostridium perfringens, Enterococcus faecalis* JH2-2 and *Enterococcus faecalis* V583. Δ*bac*: *Ent. faecalis* 14 mutant deleted in *ddA* and *ddB* bacteriocin structural genes, Δ*bac*-Comp: the Δ*bac* complemented strain, Δ*ddI*: *Ent. faecalis* 14 mutant deleted in *ddI* gene, Δ*ddI-Comp*: the Δ*ddI* complemented strain, Δ*ddI*Δ*bac*: *Ent. faecalis* 14 mutant deleted in *ddI, ddA*, and *ddB* genes. If present, the inhibition zone indicated the susceptibility of the bacterial lawn (target bacteria) to the produced EntDD14 bacteriocin. The data are representative of at least three independent experiments.

The antibacterial activity of *Ent. faecalis* 14 against *L. innocua, C. perfringens, Ent. faecalis* JH2-2 and *Ent. faecalis* V583 was solely attributable to EntDD14, and not to any other potential antibacterial agent, which could be produced by the WT strain. Indeed, the Δ*bac* mutant strain, deleted in *ddA* and *ddB* genes, was completely devoid of any antibacterial activity despite the comparable pH values of the supernatants obtained from the mutant and the WT strains. Noteworthy, after 24 h of culture, the supernatant final pH measured was 5.12 for the WT strain and 4.87 for the Δ*bac* mutant strain. This slight difference in pH values could be explained by the basic nature of the two peptides of EntDD14 produced by the WT strain. Similar data were obtained with the WT vs. the Δ*ddI*Δ*bac* triple mutant strain (Figure 4), which strengthens this argument. Trans-complementation assays conducted upon cloning the *ddA* and *ddB* genes into the Gram-positive replicative plasmid pAT18 recovered the WT phenotype in the Δ*bac* mutant strain as shown by the complemented strain *Ent. faecalis 14* Δ*bac-Comp* phenotype. Therefore, production of EntDD14 was comparable between the WT and the *Ent. faecalis 14* Δ*bac-Comp* complemented strains (Figure 4). Thus, the inhibition potency of *Ent. faecalis* 14 was exclusively due to EntDD14 production, and not to any other factor such as pH decrease or organic acid production, which occur in LAB during glucose metabolism.

### The Role of the ABC Transporter in the Transport of EntDD14

Bacterial ABC transporters are often composed of an ATP-binding protein and one or more accessory proteins (Beis and Rebuffat, 2019). In the EntDD14 locus, the ABC transporter is located downstream of the structural genes encoding for the EntDD14 enterocin. As shown in Figure 1 and Table 4, the ABC transporter associated with EntDD14 production contained at least three genes named *dd-H, I*, and *J*. The gene *ddH* encodes a putative efflux system protein YvrP. The *ddI* gene encodes an ABC transporter ATP-binding protein of the ATPase family, and the *ddJ* gene encodes a putative macrolide export ATP-binding/permease protein (MacB). Our results show that this ABC transporter is involved in the active transport of intracellular EntDD14 peptides to the outside of the membrane. Indeed, the mutant deleted in the *ddI* gene encoding an ATP binding cassette of the ABC transporter has considerably reduced the production of EntDD14, comparative to the WT strain (Figure 4). Indeed, the Δ*ddI* mutant strain produced only 25% of the EntDD14 produced by the WT strain. This rate was based on the anti-*Listeria* activity of the Δ*ddI* mutant strain (200 AU/mL) compared to the WT (800 AU/mL). The levels of EntDD14 production were restored when the Δ*ddI* mutant strain was complemented with a functional *ddI* gene cloned into pAT18 vector (*Ent. faecalis* Δ*ddI-Comp*) (Figure 4), arguing the role of this putative transporter in the active export of EntDD14. Nevertheless, this result suggested that additional systems are also exporting EntDD14 outside of the cell, as this mutant strain, was still producing about 25% of EntDD14 compared to the WT strain. To check whether other non-specific enterococcal transporters were involved in EntDD14 export, we transformed the non-bacteriocinogenic and sensitive *Ent. faecalis* JH2-2 strain with the EntDD14 precursor including its promoter region and structural *ddA* and *ddB* genes cloned into the pAT18 vector. No production of EntDD14 was observed in the resulting strains (data not shown).

### The Contribution of the ABC Transporter to the Immunity System Against Exogenous EntDD14

**Figure 5:**
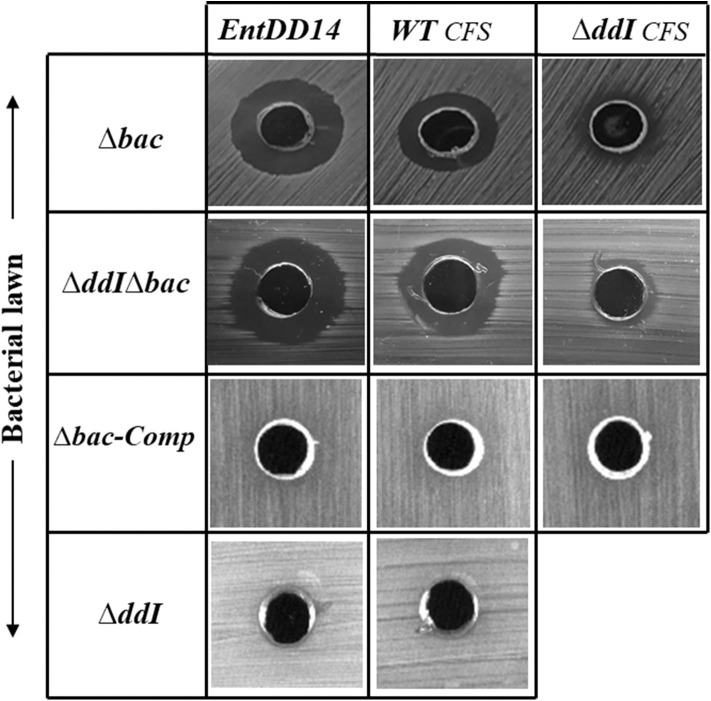
Sensitivity of the *Ent. faecalis* 14 mutant and complemented strains to pure EntDD14 at 60 μg/mL, cell free supernatant (CFS) of the wild-type strain and that of the Δ*ddI* mutant. Δ*bac*: *Ent. faecalis* 14 mutant deleted in *ddA* and *ddB* bacteriocin structural genes, Δ*ddI*Δ*bac*: *Ent. faecalis* 14 mutant deleted in *ddI, ddA*, and *ddB* genes, Δ*bac*-Comp: the Δ*bac* complemented strain and Δ*ddI*: *Ent. faecalis* 14 mutant deleted in *ddI* gene. If present, the inhibition zone indicates the susceptibility of the bacterial lawn (target bacteria) to EntDD14. The data are representative of at least three independent experiments.

**Figure 6:**
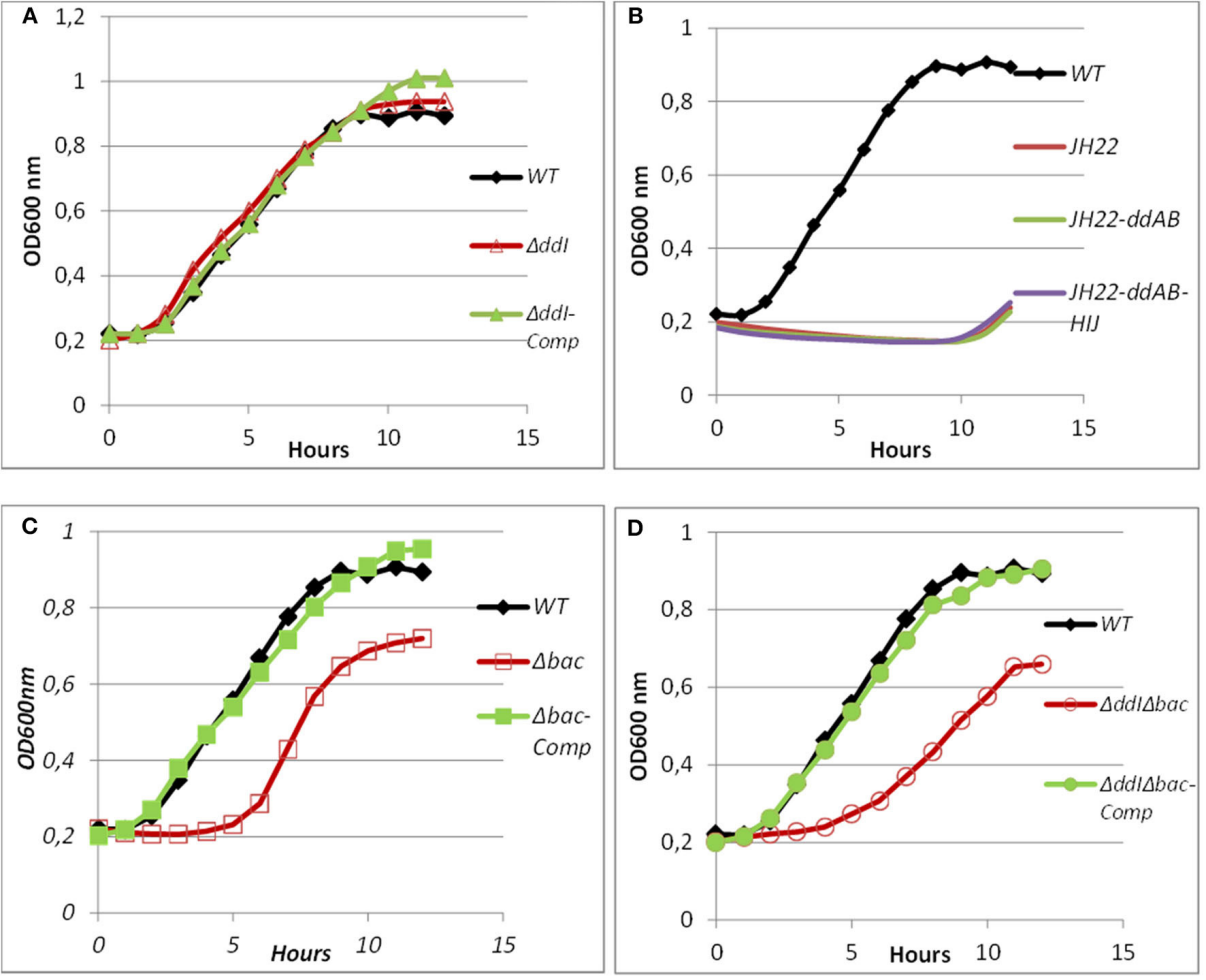
Growth curves of WT, mutated and complemented *Ent. faecalis* 14 strains in the presence of pure EntDD14 at 10 μg/mL. *Ent. faecalis* 14 WT (♦) **(A)**: Δ*ddI* mutant (Δ) and the Δ*ddI* complemented strain (▴); **(B)**: *Ent. faecalis JH2-2* (red line) *Ent. faecalis* JH2-2*-ddAB* (green line) and *Ent. faecalis JH2-2-ddAB-HIJ* (purple line); **(C)**: Δ*bac* mutant (□) and the Δ*bac* complemented strain (■); and **(D)**: Δ*ddI*Δ*bac* mutant (°) and the Δ*ddI*Δ*bac* complemented strain (•).The data are the means of at least three independent experiments.

ABC transporters can contribute to bacteriocin immunity by pumping bacteriocins to the outside of the cell membrane (Diaz et al., 2003; Sánchez-Hidalgo et al., 2003; Beis and Rebuffat, 2019). To better understand the role of the ABC transporter in immunity to EntDD14, we performed sensitivity tests to EntDD14 by the well-diffusion method and also by growth kinetics in the presence of purified EntDD14 (10 μg/mL). Unexpectedly, the Δ*ddI* mutant strain did not exhibit any sensitivity to EntDD14 even at highest concentration (60 μg/ml) nor to the cell-free supernatant (CFS) obtained from the WT strain (Figure 5). Notably, EntDD14's MIC values were similar to those obtained for the WT (resistant phenotype). Growth kinetics in the presence of EntDD14 at 10 μg/mL showed no significant differences between the WT strain, the Δ*ddI* mutant strain and the complemented *ddI-Comp* strain (Figure 6A). These results refuted therefore the involvement of an ATP-binding protein/ATPase transporter in the self-immunity system of EntDD14. In order to unravel the role of the entire ABC transporter (*ddHIJ*) in the mechanism of immunity, we constructed a recombinant plasmid pAT18:*ddAB-HIJ* which we used to transform the sensitive *Ent. faecalis* JH2-2 strain. In this case, if the ABC transporter played a dual transport and immunity function, then this construction would be sufficient to produce EntDD14 and to immunize the host strain against the inhibitory action of EntDD14. Unexpectedly, the obtained clones of *Ent. faecalis* JH2-2-*ddAB-HIJ* were unable to produce EntDD14. In addition, growth kinetics in the presence of 10 μg/mL of EntDD14 showed the sensitive phenotype of *Ent. faecalis* JH2-2 compared to the WT strain. Overall, no significant difference was observed between *Ent. faecalis* JH2-2 and the transformed strain, JH2-2-*ddAB-HIJ* (Figure 6B). To verify the integrity of the pAT18:*ddAB-HIJ* construction, we used it in order to complement the Δ*bac* double mutant strain, which is deleted in both *ddA* and *ddB*, and also to transform the Δ*ddI*Δ*bac* triple mutant strain, which is deleted in genes *ddA, ddB* and *ddI* of the ABC transporter. Remarkably, the WT phenotype was restored in both cases. Indeed, the pAT18:*ddAB-HIJ* construction transformed the Δ*bac* mutant strain to a producer strain and the resulting transformed strain Δ*ddI*Δ*bac* produced as much EntDD14 as the WT (data not shown).

All these data ruled out the involvement of the ABC transporter in the primary self-immunity system of the two peptides leaderless bacteriocin EntDD14. Moreover, the precursor genes *ddAB* and ABC transporter genes *ddHIJ* in themselves seem to be insufficient to produce EntDD14.

### Intracellular EntDD14 Is Involved in the Self-Immunity System

Mutants obtained during this study were screened for their susceptibility to bacteriocin EntDD14. Of importance, the Δ*bac* mutant strain appeared to be sensitive to its own bacteriocin. This was observed not only when the mutant strain was treated with pure EntDD14 at 60 μg/mL but also when it was exposed to the supernatant of the WT strain, or to that of the less producing strain Δ*ddI* (Figure 5). During growth in the presence of 10 μg/ml of EntDD14, the Δ*bac* mutant strain extended its lag phase and entered the exponential growth phase only after 5 h of incubation (Figure 6C). The OD_600_ values obtained after 12 h of growth were significantly different between the WT strain and the Δ*bac* mutant strain (*P* = 0.0001). The trans-complementation experiment using the pAT18-*ddAB* construction allowed the Δ*bac* mutant strain to recover its resistance to EntDD14 (Figures 5, 6C). To confirm this result, we constructed a Δ*bac* mutant in a host strain other than *Ent. faecalis* 14, and thus selected the *Ent. faecalis* 14 Δ*ddI* mutant strain. This strain was resistant to EntDD14 and produced about 25% of this bacteriocin compared to the WT strain. As expected, Δ*ddI*Δ*bac* did not produce EntDD14 and was found to be sensitive to this bacteriocin, like the Δ*bac* mutant strain (Figures 5, 6D). The complementation of this triple mutant with the pAT18:*ddAB-HIJ* construction fully restored the phenotype of the WT strain, which displayed almost identical kinetics in the presence of EntDD14 at 10 μg/mL (Figure 6C). Taken together, these results highlight the clear role of intracellular EntDD14 in the mechanism of its own immunity.

### Gene Expression Analysis of EntDD14 Cluster

**Figure 7:**
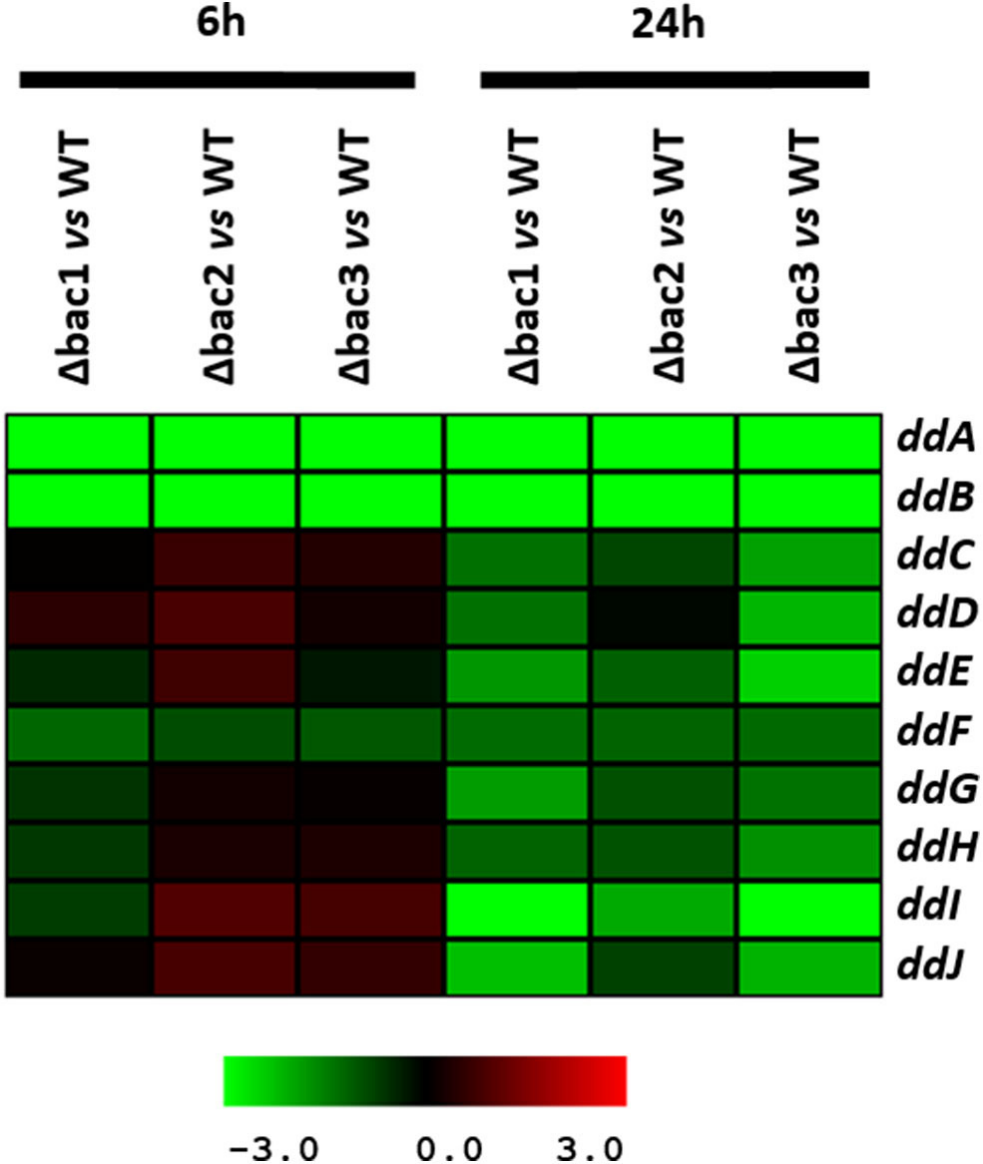
Profile of the EntDD14 predicted ORFs in Δ*bac vs*. WT strain. Log2 ratios were registered for three biological repetitions after 6 and 24 h of growth in GM17 medium.

To explain the sensitive phenotype of the Δ*bac* mutant strain to EntDD14, we checked whether the genes *ddCDEFGHIJ* were expressed in this strain. To this end, we used data generated from a comparative transcriptomic analysis of the Δ*bac* mutant strain vs. the WT, after 6 and 24 h of growth in GM17 medium. The gene expression analysis did not reveal any significant differences between these strains after 6 h of growth except for the *ddF* gene which was slightly down-regulated, as the log2 ratio Δ*bac*/WT ranged from −1 to 1 (Figure 7 and Table S1). Interestingly, all analyzed *ddCDEFGHIJ* genes were found to be significantly down-regulated in the Δ*bac* mutant strain after 24 h of growth in GM17 (Figure 7 and Table S1). Moreover, mean gene expression showed that *ddI, ddE* and *ddJ* genes were the most deregulated with log2 Δ*bac*/WT ratios of − 2.86, − 1.78, and − 1.70, respectively (Table S1). Finally, as expected, *ddA* and *ddB* encoding the structural genes were highly down-regulated in the Δ*bac* mutant strain at both 6 and 24 h since they were deleted in this host (Figure 7 and Table S1).

## Discussion

Bacteriocins are known for their effectiveness in fighting and eradicating microbial pathogens (Quintana et al., 2014; Graham et al., 2017; Johnson et al., 2018). Interactions of these charged antimicrobial peptides with cell membrane leads to the formation of ion-permeable channels, resulting in perturbation of ion gradients by passive efflux of vital intracellular metabolites, such as ions, amino acids and ATP, which causes serious metabolic disruption and finally cell death (Karpinski et al., 2016). At the molecular level, bacteriocin production requires complex machinery consisting of the transcription-translation processes, transport out of the cell, and also immunity mechanisms to protect the strain from the toxicity of its own products (Bastos et al., 2015; Bountra et al., 2017).

Leaderless bacteriocins are active after their translation, and do not undergo significant post-translational modifications or processing. Therefore, bacteria producing such bacteriocins have certainly specific immunity systems that ensure their protection from the toxicity of their own peptides. This protection is expected to be ascertained from the translation step until their export outside of the cell. Of note, no-dedicated immunity proteins have been reported for leaderless two-peptide bacteriocins. Only the role of export and immunity of the ABC transporter has been described. Several mechanisms of self-immunity have been described for many bacteriocins. These involve for the well-known nisin, a lipoprotein, designed NisI, which is able to bind and thereby prevent nisin to access its predicted target lipid II through direct interactions (Hacker et al., 2015). In direct line, Abi proteins, which are putative membrane-bound metalloproteases, have been reported to play a role in the self-immunity of bacteriocins (Kjos et al., 2010). Using EntDD14 as a representative model of this category of bacteriocins, based on its high homology with other known leaderless two peptides bacteriocins (Figure 3), we have undertaken a genetic study to obtain relevant information on the immunity system of two-peptide leaderless bacteriocin-producers. First, an *in silico* genetic analysis of the EntDD14 bacteriocin region predicted a least 10 ORFs (*ddABCDEFGHIJ*), which could be organized into three closed operons. As shown in Figure 2, predictions of mRNA folding, using ARNold and RibEX algorithms permitted the location of a potential secondary structure between *ddB and ddC*, suggesting a possible post-transcriptional regulation, in which the synthesis of peptides A and B can be dissociated from the translation of other genes. To the best of our knowledge regulation and expression of bacteriocins at the post-transcriptional level has been reported only for the cyclic Enterocin AS-48 (Fernández et al., 2008).

Here, a BLAST analysis of the proteins encoded by the EntDD14 gene cluster did not reveal any significant homology with other known dedicated immunity proteins. However, sequence alignment established that the *ddHIJ* genes share significant similarities with several of the ABC transporters found in most leaderless, and cyclic bacteriocins, despite the absence of sequence similarities for the structural genes encoding these bacteriocins (Cintas et al., 2000; Diaz et al., 2003; Iwatani et al., 2012; Nascimento et al., 2012). These data support a potential relationship in the mediated mechanisms (production and/or immunity) and a close structural feature between leaderless and cyclic bacteriocins (Towle and Vederas, 2017; Perez et al., 2018). In the case of EntDD14, the ABC transporter was found to be involved in bacteriocin export, as a Δ*ddI* mutant strain, affected in an ATP-binding protein of the ATPase family, could produce only 25% of bacteriocin compared to the wild-type strain. Similar results were reported for the leaderless single peptide aureocin 53. Indeed, the aureocin 53 bacteriocinogenic strain could produce only 24% of this bacteriocin when the ABC transporter *aucEFG* was affected in the first gene *aucE* (Nascimento et al., 2012). Here, we established that in the Δ*ddI* mutant, production of EntDD14 was not completely abolished, indicating that another export pathway is also used by the bacteriocinogenic strain. Unexpectedly, the susceptible *Ent. faecalis* JH2-2 strain was unable to produce EntDD14 when transformed by the *ddAB* genes. In terms of the bacteriocin export pathways, it was established that enterocin L50 can be successfully expressed heterologously in two yeast expression systems through the general secretory pathways (Basanta et al., 2009, 2010), suggesting a possible use of another transport pathway. Here, the *ddF* gene, which encodes a putative pH domain protein, was found to be down-regulated after 6 h and 24 h of growth in the Δ*bac* mutant but not in the wild-type strain (Figure 7, Table S1). The *ddF* gene displayed 23% identity (and 42% of positive residue) with *orf8* of the pRJ9 plasmid of *S. aureus* (Netz et al., 2002). Remarkably, in *S. aureus*, the disruption of this gene abolished bacteriocin production (Nascimento et al., 2012). Therefore, we can hypothesize that *ddF* gene might play a role in the export of EntDD14.

The three-component ABC transporters, which are similar to those of *ddHIJ*, were previously shown to contribute to bacteriocin immunity by active extrusion of the peptide, most likely by reducing the concentration of the bacteriocin in direct contact with the cytoplasmic membrane (Klein and Entian, 1994; Siegers and Entian, 1995; Yarmus et al., 2000). Similarly, Rincé et al. (1997) reported that the self-immunity to the lantibiotic lacticin 481 was abolished when any *lctE, lctF*, or *lctG* gene was deleted (Rincé et al., 1997).

Taking into consideration all these published data, and in order to gain novel insights into the immunity systems of two-peptide leaderless bacteriocins, we looked at the role of the ABC transporter in the synthesis and immunity of EntDD14. To this end, we tested the sensitivity of the Δ*ddI* mutant strain to EntDD14, and also by cloning *ddAB-HIJ* genes and studying their effects in the sensitive *Ent. faecalis* JH2-2 strain. Consequently our data were in discordance with those formerly reported. Indeed, the Δ*ddI* mutant strain, as well as the WT strain were resistant to EntDD14 (up to 60 μg/mL) (Figures 5, 6A). Furthermore, cloning and expression of the full ABC transporter in *Ent. faecalis* JH2-2 strain did not grant any resistance to EntDD14 even at low concentrations (Figure 6B). Although cloning of the entire ABC transporter did not allow the *Ent. faecalis* JH2-2 strain to gain resistance to exogenous EntDD14, this does not necessarily rule out the role of this ABC transporter in the detoxification of intracellular EntDD14. This has already been attributed to the ABC transporter for aureocin 53, which contains two other immunity proteins named AucIA and AucIB (Nascimento et al., 2012). AucIA and AuIB exhibited, respectively, 28 and 29% identity with the proteins encoded by *ddD* and *ddC*. These homologies were even higher, 52 and 60%, when compared to the products of DdC and DdD, and their aureocin counterparts AucIA and AucIB, suggesting therefore that the DdC and DdD proteins could be good candidates for studying the immunity of bacteriocinogenic *Ent. faecalis* 14.

Considering the different genetic constructions, their expression and phenotypes, we assume that intracellular EntDD14 plays a role in its own immunity system. This original finding was strengthened by the phenotypes of the Δ*bac* and Δ*ddI*Δ*bac* mutant strains, which exhibited sensitivity to EntDD14, as opposed to the WT and the complemented strains (Figure 5). At low EntDD14 concentration, the Δ*bac* and Δ*ddI*Δ*bac* mutant strains showed an extended lag phase and a final OD_600_ significantly below that of the WT strain (Figures 6C,D), pointing again to the role of the intracellular EntDD14 in its own immunity system. The sensitivity of null-mutant strains was not ascribed to shut-down of expression of the *ddCDEFGHIJ* genes, as mostly these genes were expressed during the log phase of the Δ*bac* mutant strain (Figure 7, Table S1).

The comparative study of the gene expression of the Δ*bac* mutant strain vs. WT strain underpinned the role of EntDD14 in the positive regulation of the *ddCDEFGHIJ* genes since all of them were significantly down-regulated in the Δ*bac* mutant strain, mainly after 24 h of growth (Figure 7, Table S1). Recent studies have reported the implication of transcriptional regulators in the synthesis of lacticin Q and aureocin A70 (Coelho et al., 2016; Iwatani et al., 2016). Environmental factors such as temperature or nutrition-adaptation were reported to control biosynthesis of enterocin L50 and enterocin Q in the multiple bacteriocin producing strains *Ent. faecium* L50 (Criado et al., 2006) and *Weissella hellenica* QU 13 (Masuda et al., 2016). In the case of non-leaderless bacteriocins, regulation by quorum sensing and leader peptide seemed to be involved (Kleerebezem, 2004; Kuipers et al., 2004; Rink et al., 2007; Abts et al., 2013). Related to that, Perez et al. (2017) reported that mutations in the leader peptide altered the overall conformation of the precursor peptide, which reduced, or enhanced its ability to fit and interact with the substrate-binding cleft of its processing enzyme(s).

As a conclusion, we report that to the best of our knowledge, an intracellular two-peptide leaderless bacteriocin plays a role in its own self-immunity system. The characterization of other genes of the EntDD14 operon (*ddCDEFG*) and their role in overall EntDD14 expression and immunity constitutes our next goal.

## Data Availability Statement

The datasets presented in this study can be found in online repositories. The names of the repository/repositories and accession number(s) can be found in the article/Supplementary Material.

## Author Contributions

DD, AB, and RL conceived the ideas, designed the experiments, discussed the data throughout the project, and wrote the article. RL performed the experiments and AL-D did the transcriptomic analysis. DD, RL, AB, and AL-D revised and approved the manuscript dissertation. All authors contributed to the article and approved the submitted version.

## Conflict of Interest

The authors declare that the research was conducted in the absence of any commercial or financial relationships that could be construed as a potential conflict of interest.
