# Supplementary material for: A Leaderless Two-Peptide Bacteriocin, Enterocin DD14, Is Involved in Its Own Self-Immunity: Evidence and Insights
Source: Front Bioeng Biotechnol. 2020 Jun 26;8:644. doi: 10.3389/fbioe.2020.00644 (PMC7332713; doi:10.3389/fbioe.2020.00644)
Supplement: Supplementary file 1 [file Data_Sheet_1.pdf]

Supplementary data

A Leaderless Two-Peptide Bacteriocin Enterocin DD14 is Involved in its Own Self-Immunity: Evidence and Insights

Rabia Ladjouzi[1], Anca Lucau-Danila[1], Abdellah Benachour[2], Djamel Drider[1]*

[1] UMR Transfrontalière BioEcoAgro N° 1158, Univ. Lille, INRAE, Univ. Liège, UPJV, YNCREA, Univ. Artois, Univ. Littoral Côte d'Opale, ICV – Institut Charles Viollette, F-59000 Lille, France. [2] UR Risques Microbiens, Normandie Univ, UNICAEN, U2RM, 14000 Caen, France.

Figure S1. Mutagenesis strategy for the construction of *Ent. faecalis* 14 *ΔddAΔddB* mutant.

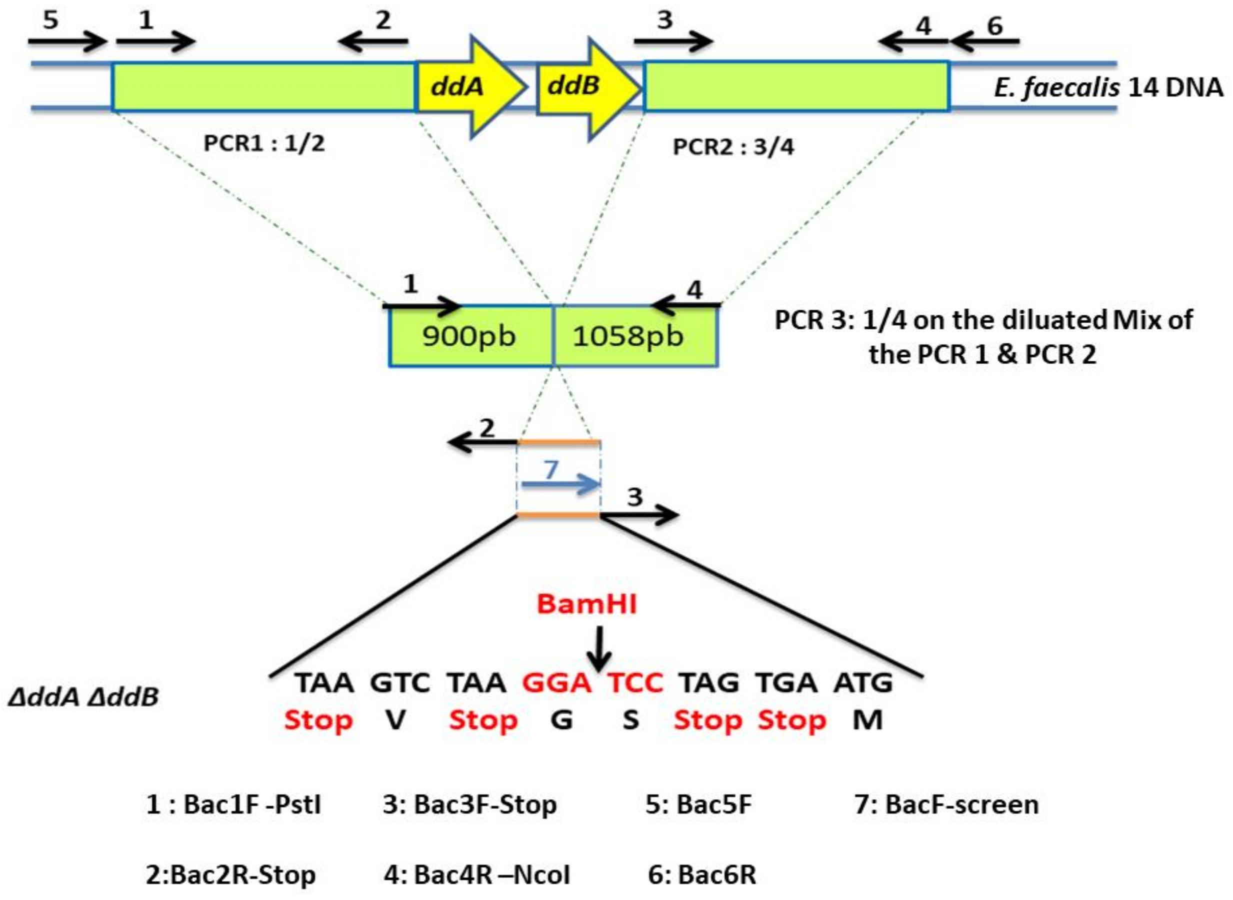

Supplementary data

Table S1. Profile of DD14 predicted ORFs in *Ent. faecalis* 14 *Δbac* mutant versus WT strain. Log2 ratio were registered for three biological repetitions after 6h and 24h of growth in GM17 medium.

		**6 hours of growth**				**24 hours of growth**			
**Gene name**	**Microarray ID**	*Δbac*1-6h/WT1	*Δbac* 2-6h/WT2	*Δbac* 3-6h/WT3	**Mean At 6H**	*Δbac* 1-24h/WT1	*Δbac* 2-24h/WT2	*Δbac* 3-24h/WT3	**Mean At 24H**
ddA	CUST_250_PI442896112	**-6,4196***	**-7,3822***	**-7,3456***	**-7,0491***	**-5,0902***	**-5,1998***	**-5,1998***	**-5,1633***
ddB	CUST_251_PI442896112	**-7,5043***	**-8,3918***	**-8,3918***	**-8,0960***	**-6,3507***	**-6,4951***	**-6,6137***	**-6,4865***
ddC	CUST_252_PI442896112	0,0570	0,6852	0,4266	0,3896	**-1,3377***	-0,8344	**-1,8949***	**-1,3557***
ddD	CUST_3_PI443061160	0,5161	0,8684	0,2238	0,5361	**-1,3357***	-0,0802	**-2,1416***	**-1,1858***
ddE	CUST_4_PI443061160	-0,4772	0,7640	-0,2595	0,0091	**-1,7693***	**-1,1285***	**-2,4560***	**-1,7846***
ddF	CUST_5_PI443061160	**-1,2142***	-0,9058	**-1,0266***	**-1,0489***	**-1,2656***	**-1,1648***	**-1,2503***	**-1,2269***
ddG	CUST_6_PI443061160	-0,6088	0,2325	0,0958	-0,0935	**-1,8391***	-0,9509	**-1,3434***	**-1,3778***
ddH	CUST_7_PI443061160	-0,6561	0,3194	0,3450	0,0028	**-1,1705***	-0,9744	**-1,6994***	**-1,2814***
ddI	CUST_255_PI442896112	-0,6977	0,9672	0,8348	0,3681	**-3,2978***	**-2,0092***	**-3,2786***	**-2,8619***
ddJ	CUST_256_PI442896112	0,1264	0,8549	0,6154	0,5323	**-2,2415***	-0,7703	**-2,1176***	**-1,7098***

(*) significantly down-regulated in *Δbac* mutant strain
